# Effect of Coronary Artery Disease on COVID-19—Prognosis and Risk Assessment: A Systematic Review and Meta-Analysis

**DOI:** 10.3390/biology11020221

**Published:** 2022-01-29

**Authors:** Lukasz Szarpak, Malgorzata Mierzejewska, Jonasz Jurek, Anna Kochanowska, Aleksandra Gasecka, Zenon Truszewski, Michal Pruc, Natasza Blek, Zubaid Rafique, Krzysztof J. Filipiak, Andrea Denegri, Milosz J. Jaguszewski

**Affiliations:** 1 Institute of Outcomes Research, Maria Sklodowska-Curie Medical Academy, 03-411 Warsaw, Poland; 2 Research Unit, Maria Sklodowska-Curie Bialystok Oncology Center, 15-026 Bialystok, Poland; 3 1st Chair and Department of Cardiology, Medical University of Warsaw, 02-091 Warsaw, Poland; mmierzejewska97@gmail.com (M.M.); jurekjonasz@gmail.com (J.J.); s073863@student.wum.edu.pl (A.K.); gaseckaa@gmail.com (A.G.); 4 Department of Emergency Medicine, Medical University of Warsaw, 02-005 Warsaw, Poland; ztruszewski@wum.edu.pl; 5 Research Unit, Polish Society of Disaster Medicine, 05-806 Warsaw, Poland; m.pruc@ptmk.org; 6 Institute of Clinical Medicine, Maria Sklodowska-Curie Medical Academy, 04-311 Warsaw, Poland; natasza.blek@gmail.com (N.B.); krzysztof.filipiak@uczelniamedyczna.com.pl (K.J.F.); 7 Henry JN Taub Department of Emergency Medicine, Baylor College of Medicine, Houston, TX 77030, USA; zubaidrafique@gmail.com; 8 Cardiology Division, Department of Biomedical, Metabolic and Neural Sciences, University of Modena and Reggio Emilia, Policlinico di Modena, 41121 Modena, Italy; denegriandrea@msn.com; 9 1st Department of Cardiology, Medical University of Gdansk, 80-294 Gdansk, Poland; jamilosz@gmail.com

**Keywords:** coronary artery disease, CAD, COVID-19, novel coronavirus, systematic review

## Abstract

**Simple Summary:**

As of October 2021, over 245 million people have been infected and nearly 5 million have died due to COVID-19. Atherosclerosis is a lipid-driven chronic inflammation of the arterial wall with coronary artery disease (CAD) that may lead to acute coronary syndrome (ACS), which remains the main cause of death in developed countries. We believe that due to CAD development factors affecting the vessel wall, the SARS-CoV-2 infection may precipitate further advancement of future thromboembolic events by a plaque erosion or rupture. Therefore, this metanalysis aims to unambiguously establish the role of the history of CAD in mortality and severity of COVID-19 disease.

**Abstract:**

Coronary artery disease (CAD) is the leading cause of death worldwide. Patients with pre-existing CAD were shown to have a more severe course of COVID-19, but this association has not been clarified. We performed a meta-analysis to determine the association between CAD and COVID-19 outcomes. We searched Scopus, Medline (PubMed), Web of Science, Embase, and Cochrane databases up to 2 November 2021. There were 62 studies with a total population of 49,286 patients included in the meta-analysis. CAD occurrence in survivor vs. non-survivor groups varied and amounted to 9.2% vs. 22.9%, respectively (OR = 0.33; 95%CI: 0.29 to 0.39; I^2^ = 70%; *p* < 0.001). CAD was also associated with increased severity of COVID-19 disease and was (10.8% vs. 5.6%, respectively, for severe vs. non-severe groups (OR = 2.28; 95%CI: 1.59 to 3.27; I^2^ = 72%; *p* < 0.001). The role of history of CAD in mortality and severe condition in COVID-19 presents itself as prominent—although a risk of bias in retrospective trials needs to be assessed, in case of our meta-analysis the statistically significant results when it comes to higher mortality among patients with CAD compared to non-CAD patients, a more severe condition observed in patients with CAD, and a visibly more frequent admission to intensive care unit in patients with CAD, it seems that an incidence of cardiovascular events plays a role in COVID-19 prognosis.

## 1. Introduction

COVID-19, caused by new coronavirus SARS-CoV-2, was first observed in Wuhan, China in December 2019 [1]. Within three months it spread all around the world achieving the status of a pandemic on 11th March 2020 [2]. As of 17 November 2021, 254,589,111 people have been infected and 5,118,866 have died due to COVID-19 [3].

Over the last two decades, the world population has experienced four major outbreaks of coronaviruses. In 2002 in China, SARS-CoV-1 infected 8000 people, killing almost 10% of them. Thereafter, MERS-CoV spread in Saudi Arabia (2012) and Korea (2015), affecting 2000 people with approximately 35% mortality [4]. The fourth outbreak was caused by SARS-CoV-2 and resulted in the current pandemic. The manifestations of COVID-19 are not specific. The most common symptoms are cough, fever, tiredness and loss of smell and taste, but production of sputum, sore throat, headache, nausea vomiting and diarrhea may also indicate infection [1,5,6,7]. Transmissibility peaks just before their onset. Among individuals without any sign of illness, it may sustain for even 21 days [8]. Men are at higher risk of severe outcome of the disease. Male sex associates also with higher case-fatality [9,10,11]. In some patients, the disease may progress to acute respiratory distress syndrome (ARDS), whereas in others it may never manifest any symptoms. It is essential that even in asymptomatic patients abnormalities including cardiac magnetic resonance features of myocarditis could be found. Moreover, long-term consequences, such as fatigue, muscle weakness, sleep difficulties, anxiety and depression were described. It was suggested that fatigue and weakness may in some cases derive from developing cardiac dysfunction [7]. Vaccination is the only effective method that reduces morbidity and mortality rates as well as the risk of long-term complications [12].

Coronary artery disease (CAD) remains the leading cause of death worldwide. According to the Global Burden of Disease 2015 Study (GBD 2015), in 2015 it affected over 110 million people and has taken about 8.9 million human lives [11,12]. An atherosclerotic plaque, which accumulates in epicardial arteries, is no longer believed to be only a hemodynamic issue. Currently, the inflammatory component of the disease is acknowledged. Moreover, inflammation is considered to participate both in local and systemic consequences of atherosclerosis [13]. The pathophysiology of coronary atherosclerosis involves many agents such as leukocytes, smooth muscle cells, metalloproteinases and a whole range of proinflammatory cytokines and complement components [13]. The calcification of the vessel wall is one of the consequences of this process. The clinical presentation of the CAD varies and can be described as either acute coronary syndrome (ACS) or chronic coronary syndrome (CCS). Due to the atherosclerotic plaque rupture or erosion, that induces an atherothrombotic event, an ACS may occur [14].

Underlying comorbidities, particularly cardiovascular diseases, have been associated with worsened outcomes of COVID-19 patients [15,16]. Since coronary artery disease (CAD) reduces oxygen supply to the myocardium, a respiratory infection or pneumonia may decrease oxygenation capacity and further exacerbate the insufficient oxygen delivery to the heart. Prior studies suggest that CAD aggravates the course of COVID-19, exacerbating hypoxemia and leading to severe disease [17]. Furthermore, atherosclerotic plaque is widely known to be a source of immune system dysregulation and a chronic inflammatory state which combined with diffuse endothelitis may lead to multiorgan damage in the course of a COVID-19 disease. It is also assumed that a more aggressive viral replication can therefore be induced [18]. Here, we performed a meta-analysis of evaluation of the association between CAD and COVID-19 outcomes.

## 2. Materials and Methods

The current trial was designed as a systematic review and meta-analysis and conducted following the Cochrane Handbook for Systematic Reviews [19]. The results are reported in accordance with the Preferred Reported Items for Systematic Reviews and Meta-Analysis (PRISMA) statement [20]. A protocol of this meta-analysis has not been registered. Due to the nature of the study, the consent of the bioethical committee was not required.

### 2.1. Literature Search

A literature search was carried out using Medline (PubMed), Embase, Scopus, Web of Science and Cochrane Central Register of Controlled Trials (CENTRAL) databases from inception to 2 November 2021. Articles in English were included in the research. No exclusions were made for disease severity, or outcomes reported. Database searches were performed using the following keywords: ‘coronary artery disease’ and its synonyms (CAD, coronary vascular disease, CVD, coronary plaque, coronary vessel, coronary heart disease, left circumflex, left main, left anterior descending, right coronary) combined with the operator OR and ‘SARS-CoV-2’ and its synonyms (2019-nCoV, novel coronavirus, COVID-19) combined using the operator OR. These search terms were then combined using the operator AND. References cited in the retrieved articles were manually checked for further analysis.

### 2.2. Inclusion and Exclusion Criteria

Two reviewers (M.P. and N.B.) independently selected eligible trials. They resolved any potential disagreements by discussion with the third reviewer (L.S.). The meta-analysis research criteria were: (1) study types: prospective, retrospective, observational, cross-sectional, descriptive or case-control studies which presented data on coronary artery disease impact of COVID-19 severity; (2) patient characteristics: patients diagnosed with COVID-19 and grouped according to Diagnosis and Treatment Protocol for Novel Coronavirus Pneumonia (trial v7) from China or WHO interim guidance [21] into: moderate cases, severe cases or critical cases; survival and non-survival groups or patients required or no treatment in ICU. When there were multiple publications from the same population, only data from the most recent report was included in the meta-analysis and we excluded the rest.

### 2.3. Data Extraction

Two reviewers (A.G. and M.P.) independently performed data extraction using a purpose-designed form. They resolved potential disagreements at any stage by discussion and consensus, with residual disagreement adjudicated by a third reviewer (L.S.). The extracted data included information on: (a) study metrics: authorship, year of publication, study country of origin, study design, sample size; (b) participant characteristics: number of patients, age, male sex, occurrence of CAD, and severity outcomes. If patients’ data were missing from a study, we contacted corresponding authors for further details.

### 2.4. Risk of Bias Assessment

Two reviewers (A.G. and M.P.) independently rated all included studies for risk of bias, disagreements were resolved by a third reviewer (L.S.) if necessary. We performed a systematic assessment of bias in the included studies using the Cochrane criteria [22]. For this purpose, a tool for Risk Of Bias In Non-randomized Studies of Interventions (ROBINS-I) [23] was used. The ROBINS-I tool examines seven bias domains: (1) confounding; (2) selection of participants; (3) classification of interventions; (4) deviations from intended interventions; (5) missing data; (6) measurement of outcomes; (7) selection of the reported result. We can rate the risk of bias in each of these domains as “serious”, “moderate”, or “low”. We visualized the overall judgment of each domain using the ROBVIS tool [24].

### 2.5. Statistical Analysis

All statistical analyses were performed using the Review Manager 5.4 (Cochrane Collaboration, Oxford, UK). Descriptive statistics are presented as cases (n) and as percentages for dichotomous and categorical variables as well as means and standard deviations (SD) or median and interquartile ranges (IRQ) for continuous variables.

We quantified statistical heterogeneity on each outcome of interest using the Cochrane Q test and I^2^ statistics. Values of I^2^ statistic, ≤25%, 50%, and ≥75% indicated low, moderate, and high heterogeneity, respectively, whereas, for Q statistic, we defined substantial heterogeneity as a *p* < 0.01. When I^2^ > 50%, a random-effect model was chosen over the fixed-effect model. [25]. The Mantel–Haenszel method was used to analyze dichotomous outcomes, and we reported results as odds ratios (ORs) with 95% confidence intervals (CIs). Continuous outcome differences were analyzed using an inverse variance model with a 95%CI, and values are reported as mean differences (MDs). *p* < 0.05 was considered as significant.

## 3. Results

### 3.1. Study Characteristics

**Figure 1:**
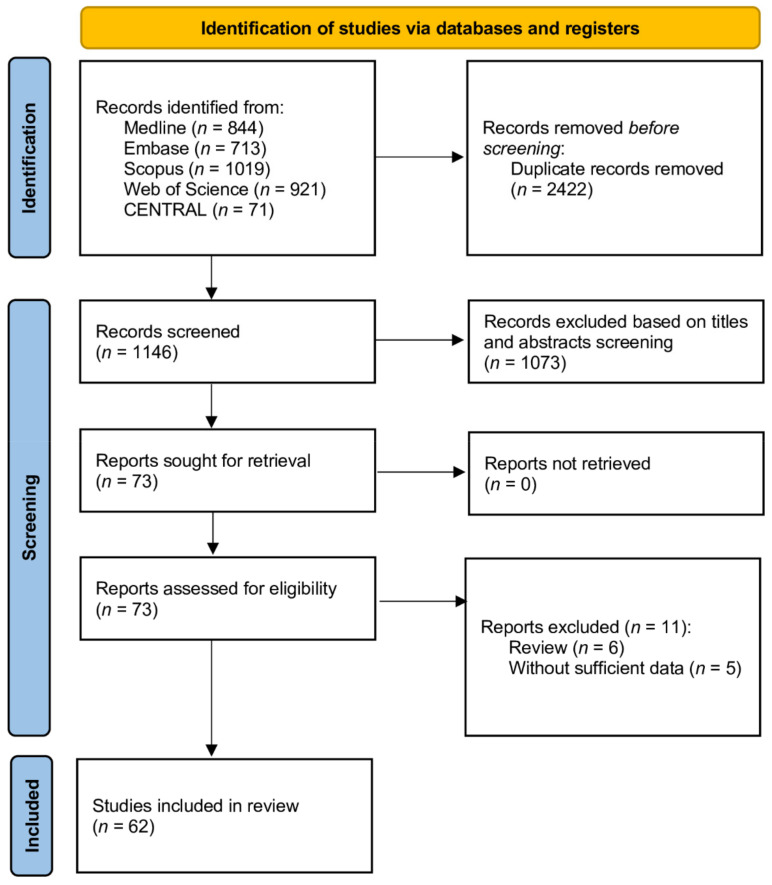
Flow diagram showing stages of database searching and study selection as per Preferred Reporting Items for Systematic reviews and Meta-analysis (PRISMA) guideline.

Following the removal of duplicates, our database search identified 1146 unique citations (Figure 1). Of these, 1073 records were excluded by screening their titles and abstracts. A full text review was conducted of the remaining 73 records, of which 11 were removed as they did not examine any association between CAD and COVID-19. Thus, 62 full articles including 49,286 patients meet the inclusion criteria and were selected to include in the review and meta-analysis. The characteristics of included studies are summarized in Table S1 in the Supplementary Materials.

The risk of bias was assessed via the ROBINS-1 tool in cohort studies assessing CAD influence on COVID-19 outcomes. Nine studies were determined to be at serious risk of bias, twenty-one moderate and thirty-two low (Figures S1 and S2).

### 3.2. Meta-Analysis

**Figure 2:**
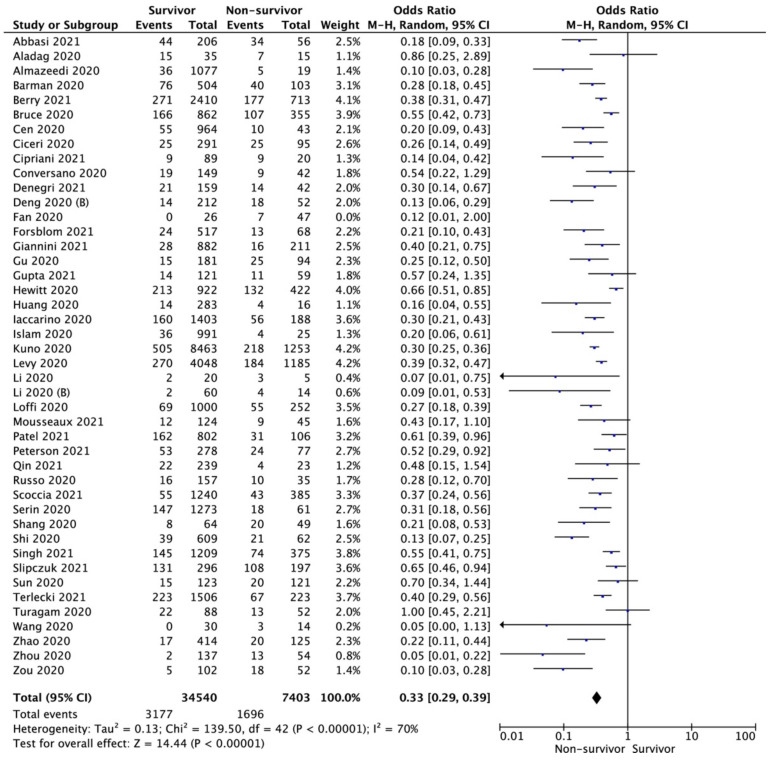
Forest plot of coronary artery disease occurrence in survivor vs. non-survivor groups. The center of each square represents the weighted odds ratios for individual trials, as the corresponding horizontal line stands for a 95% confidence interval. The diamonds represent pooled results.

Forty-three studies reported mortality among COVID-19 patients with CAD. Pooled analysis showed that CAD occurrence in survivor vs. non-survivor groups varied and amounted to 9.2% vs. 22.9%, respectively (OR = 0.33; 95%CI: 0.29 to 0.39; I^2^ = 70%; *p* < 0.001; Figure 2). In the case of excluding articles with serious risk of bias assessment, CAD occurrence in survivor vs. non-survivor groups was 8.5% vs. 21.6%, respectively (OR = 0.31; 95% CI: 0.26 to 0.36; I^2^ = 67%; *p* < 0.001).

**Figure 3:**
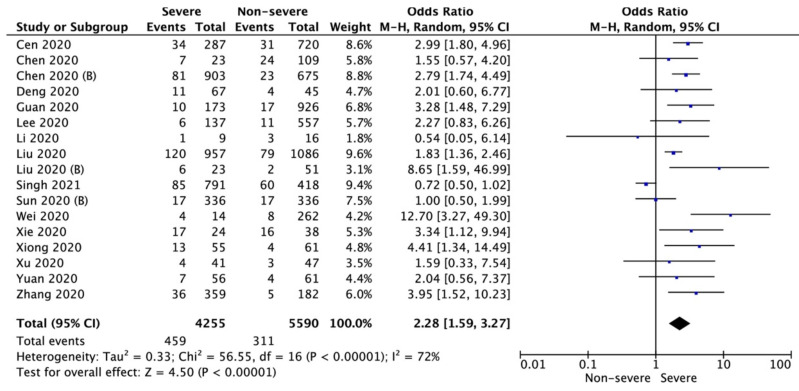
Forest plot of coronary artery disease occurrence in severe and non-severe COVID-19 patients. The center of each square represents the weighted odds ratios for individual trials, as the corresponding horizontal line stands for a 95% confidence interval. The diamonds represent pooled results.

CAD was also associated with increased severity of COVID-19 disease. Pooled analysis of seventeen studies showed that in patients with CAD severe illness was significantly higher (10.8% vs. 5.6%; OR = 2.28; 95%CI: 1.59 to 3.27; I^2^ = 72%; *p* < 0.001; Figure 3). In this case, also, no differences in the results of the analysis were observed when the articles with serious risk of bias were removed (10.2% vs. 5.3%, respectively; OR = 2.17; 95% CI: 1.46 to 3.22; I^2^ = 75%; *p* < 0.001).

**Figure 4:**
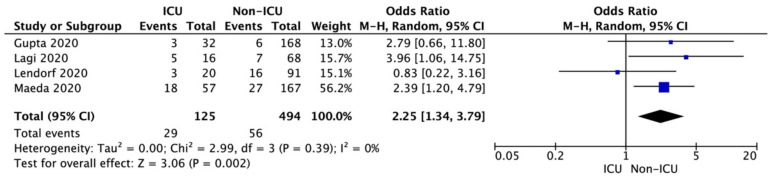
Forest plot of coronary artery disease occurrence in ICU and non-ICU group of COVID-19 patients. The center of each square represents the weighted odds ratios for individual trials, as the corresponding horizontal line stands for a 95% confidence interval. The diamonds represent pooled results.

Additionally, pooled analysis of four studies showed that patients with COVID-19 and coexisting CAD were admitted more frequently to the intensive care unit (23.2% vs. 11.3%; OR = 2.25; 95%CI: 1.34 to 3.79; I^2^ = 0%; *p* = 0.002; Figure 4).

Most of the trials were conducted as retrospective analyses. The prospective analysis design was used in 3 studies. The majority of studies were carried out on Asian and European populations, whereas several of them were held in the USA. The mean age per study ranged from 37.8 to 73.4 years.

## 4. Discussion

In this meta-analysis of 62 studies on COVID-19 possible risk factors, we tried to establish whether the incidence of CAD was associated with a more severe condition and therefore, a higher mortality in COVID-19 patients. Using a tool of assessing the risk of bias of all of our included retrospective studies, we tried to present an honest and adequate statistical significance of CAD in COVID-19, as well as a possible reasoning to those results.

Although COVID-19 commonly manifests in respiratory dysfunction, it seems that the cardiovascular system is involved more than initially expected [26]. COVID-19’s destroying action through alterations in endothelial and coagulation homeostasis may predispose to thrombosis in all arterial beds [27]. The novel coronavirus, similar to most cardiovascular risk factors, disturbs the balance between vascular relaxation and contraction as well as reduces the amount of platelet aggregation inhibitors, coagulation inhibitors and fibrinolysis activators in favor of clotting factors [28]. Venous thrombosis and pulmonary embolism are common complications of COVID-19 as a result of a disordered endothelial homeostasis triggered by either inflammatory cytokines storm or direct infection, causing, for instance, intracellular oxidative stress. [28,29]. Acute myocardial injury and CAD with thromboembolic events may be associated with insufficient endothelial function as SARS-CoV-2 infects cells responsible for vascular homeostasis [28]. Whereas vascular endothelial cells dysfunction leads to a prominent procoagulantory state due to a rapid release of high levels of cytokines associated with an infection, it is the patients with CAD and COVID-19 who present a higher risk of mortality in the patient population [27,29]. Counting hypertension, dyslipidaemia, diabetes mellitus and smoking tobacco as CAD development factors affecting the vessel wall, followed by a plaque erosion or rupture, the SARS-CoV-2 only contributes to further advancement of future thromboembolic events. Furthermore, a non-obstructive CAD along with secondary myocardial inflammation due to endothelial dysfunction has been observed [28]. Cardiac injury in COVID-19 may derive from an inflammatory factor cascade and abnormal coagulation [30]. On the other hand, direct cardiomyocyte damage, systemic inflammation and interstitial myocardial fibrosis are also probable mechanisms [28]. Therefore, without adequate treatment and secondary prevention of CAD, it seems that the interdisciplinary context of the disease ultimately leads to possible higher mortality due to numerous cardiovascular events, with COVID-19 acting as one more risk factor leading to a bad prognosis [28]. Underlying comorbidities have been associated with a higher risk of mortality among COVID-19 patients [31]. Diabetes, chronic obstructive pulmonary disease, cancer, chronic kidney failure and coronary artery disease have been listed as risk factors for disease progression [32,33,34,35]. Although many authors associated hypertension with an increased risk of mortality [32,35], Iaccarino et al. [36] did not confirm its impact on COVID-19 outcome. ECG abnormalities such as atrial fibrillation, QTc-interval prolongation and ST-segment depression found at admission were also related to increased mortality rate [37]. Another assessment of clinical risk score, named the clinical frailty score (CFS), has been associated with better predicted outcomes in the COVID-19 population than comorbidity itself [38]. COVID-19 patients with pre-existing cardiovascular disease (CVD) present the highest risk of mortality [39].

A recent study demonstrated that cardiovascular findings on computed tomography (CT) imaging performed in COVID-19 may be helpful in identifying high-risk patients who may benefit from more intense monitoring and secondary therapies [40]. Coronary artery calcification (CAC) has been associated with increased mortality in the mid-term and this finds particular significance for those with high levels of CAC such as patients with CAD [41]. What is more, patients with any CAC found in CT were more likely to be intubated and were at a higher risk of death than those without CAC, whereas in patients after coronary artery bypass graft (CABG) or stenting no such association was found. It was suggested that CAC could be used as an available, objective and complementary tool for disease risk stratification [18]. It can be obtained during non-ECG-gated low dose CT, which is used on admission in patients presenting with pneumonia in COVID-19.

CAD, indeed, has been identified as an established risk factor for severe illness and mortality in COVID-19 patients [42]. It is supposed to be one of the independent risk factors of viral RNA shedding after IgG detection, which, compared to viral clearance after IgG detection, was associated with a higher risk of severe condition during hospitalization [43]. The coexistence of other cardiovascular comorbidities, such as heart failure, exerts an independent detrimental impact on the risk of mortality in COVID-19 patients [41], which is independent of other adverse prognostic markers [44]. Cardiovascular disease burden, including acute cardiac injury, coronary artery disease, heart failure, arrhythmias have been significantly associated with mortality and intensive care admission in COVID-19 patients [45]. It is essential that further studies regarding the impact of particular cardiovascular pathologies on COVID-19 outcomes are conducted.

Currently, suitable and effective treatment is under investigation. Since the expression of the receptor for SARS-CoV-2—angiotensin-converting enzyme 2 (ACE2)—may be affected by angiotensin-converting enzyme inhibitors (ACE-I) and angiotensin receptor blockers (ARBs), their safety has been questioned. Nevertheless, the hypothesis has not been supported [36,46]. What is more, discontinuation of ACE-I, ARB, or β-blocker in patients hospitalized for a SARS-CoV-2 infection resulted in an increased risk of death [47]. Whereas medication such as ACE-I, ARBs and statins have been proven to improve endothelial function, beta-blockers, antiplatelet and/or anticoagulant drugs have been known to provide the best medical results in atherothrombotic diseases so far [27,28]. Besides reduction of oxidized low-density lipoprotein (LDL) cholesterol, among many mechanisms in which statins lead to the endothelial function improvement are increased expression of endothelial nitric oxide synthase (eNOS) and suppression of pro-oxidant enzymes and pro-inflammatory pathways [28]. Statin use has been associated with a lower risk of mortality [28,48] while no differences in terms of outcomes have been shown in pre-hospitalization use of low-dose aspirin in COVID-19 patients. Even pre-admission antithrombotic treatment, including both antiplatelet and anticoagulant medications, has not had an impact on the in-hospital mortality [35].

Our analysis is not without limitations. We assessed the risk of bias in 62 papers concerning mortality linked to COVID-19 in patients with CAD using the ROBINS-I algorithm and concluded that 32 of them were at low RoB. One of the limitations could be the variation in methodology between the studies. Only a few published studies were prospectively conducted. Thus, most studies were retrospective observational in nature and suffered limitations related to its category, including missing data related to medical history. Since all the studies consisted of patients admitted to the hospital, there is a concern of overrepresentation of severe COVID-19 cases. Several studies included only patients in intensive care units or patients who had had chest CT scans, which further limits the generalizability of their results. Only 5 out of 63 studies measured coronary artery calcification. Another limitation was related to the follow-up period. Many authors decided on arbitrary follow-up periods thus biasing the outcomes. Furthermore, there was some variation in an outcome between the studies, for instance in regard to hypertension as a risk factor of mortality. Although the studies were conducted in many countries, there is a limitation concerning examined population, which was mostly Asian and European. The fact that only articles in English were included also may influence the results. Lastly, the pandemic caused turmoil in global health and may have limited health benefits and access to health care and thus biased the outcomes and the conclusions of our study.

## 5. Conclusions

The role of CAD in mortality and severe condition in COVID-19 presents itself as prominent—although a risk of bias in retrospective trials needs to be assessed—in the case of our meta-analysis, the statistically significant results when it comes to higher mortality among patients with CAD compared to non-CAD patients, more severe conditions observed in patients with CAD, and a visibly more frequent admission to intensive care unit in patients with CAD, it seems that an incidence of cardiovascular events plays a role in COVID-19 prognosis.

## Data Availability

Source data files are available upon request.

## Supplementary Materials

The following supporting information can be downloaded at: https://www.mdpi.com/article/10.3390/biology11020221/s1, Figure S1: A summary table of review authors’ judgments for each risk of bias item for each study, Figure S2: A plot of the distribution of review authors’ judgments across studies for each risk of bias item, Table S1: Characteristics of included studies.
