# Supplementary material for: Effect of Coronary Artery Disease on COVID-19—Prognosis and Risk Assessment: A Systematic Review and Meta-Analysis
Source: Biology (Basel). 2022 Jan 29;11(2):221. doi: 10.3390/biology11020221 (PMC8868600; doi:10.3390/biology11020221)
Supplement: Supplementary file 1 [file biology-11-00221-s001.zip › biology-1493080-supplementary.pdf]

Effect of Coronary Artery Disease on COVID-19 – Prognosis and Risk Assessment: A systematic review and meta-analysis

Supplementary Material

Content:

Table S1 Characteristics of included trials. 2

Figure S1. A summary table of review authors' judgements for each risk of bias item for each study. 5

Figure S2. A plot of the distribution of review authors' judgements across studies for each risk of bias item. 6

Table S1 Characteristics of included trials.

Study	Country	Study design	Study group	No of patients	Age	Sex, male	CAD
Abbasi et al. 2021 [1].	Iran	Retrospective cohort study	Survivor	206	56 (41–65)	172 (65.6%)	44 (21.4)
			Non-survivor	56	66 (58–77)	55 (73.3%)	34 (60.7)
Aladag et al. 2020 [2].	Turkey	Retrospective cohort study	Survivor	35	68(49-75)	22 (62.3%)	15 (42.9%)
			Non-survivor	15	68(60-77)	6 (40.0%)	7 (46.7%)
Almazeedi et al. 2020 [3].	Kuwait	Retrospective cohort study	Not-ICU	1,054	37.1(16)	856 (96.4%)	33 (80.5%)
			ICU	42	54.8(11)	32 (3.6%)	8 (19.5%)
Barman et al. 2020 [4].	Turkey	Multi-center retrospective study	Survivor	504	57.5(15.4)	273 (54.2%)	76 (15.1%)
			Non-survivor	103	69.3(12.5)	61 (59.2%)	40 (38.8%)
Berry et al. 2021 [5]	USA	Retrospective cohort study	Survivor	2,410	60(48-71)	1,434 (59.5%)	271 (11.2%)
			Non-survivor	713	76(66-85)	451 (63.3%)	177 (24.8%)
Bruce et al. 2020 [6]	UK	Prospectively case report	Survivor	864	NS	484 (70.1%)	166 (60.8%)
			Non-survivor	358	NS	206 (29.9%)	107 (39.2%)
Cen et al. 2020 [7]	China	Cohort study	Non-severe	720	69 (2)	319 (44.3%)	31 (4.3%)
			Severe	287	68.6 (2.7)	174 (60.6%)	34 (11.8%)
Chen et al. 2020 [8]	China	Retrospective analysis	Non-severe	109	62.4(52.5-70)	48 (44.0%)	24 (22.0%)
			Severe	23	68.3(63-79)	15 (65.2%)	7 (30.4%)
Chen et al. 2020 (B) [9]	China	Retrospective observational study	Non-severe	675	56 (2.7)	320 (48.3%)	23 (3.4%)
			Severe	903	62.7 (3.3)	450 (49.8%)	81 (9.0%)
Ciceri et al. 2020 [10]	Italy	Retrospective analysis	Survivor	291	62(54-72)	207 (71.1%)	25 (8.6%)
			Non-survivor	95	76 (67-82)	70 (73.7%)	25 (26.3%)
Cipriani et al. 2021 [11]	Italy	Single-center, observational study	Survivor	89	69(57-79)	43 (48.3%)	9 (10.1%)
			Non-survivor	20	86(77-87)	10 (50.0%)	9 (45.0%)
Conversano et al. 2020 [12]	Italy	Retrospective, observational study	Survivor	149	60.4(13.7)	100 (67.6%)	19 (12.8%)
			Non-survivor	42	75.3(12.9)	31 (73.8%)	9 (21.4%)
Denegri 2021 [13]	Italy	Retrospective, observational study	Survivor	159	65.6(14.1)	104 (65.4%)	21 (13.3%)
			Non-survivor	42	79.7 (10.8)	25 (59.5%)	14 (33.3%)
Deng 2020 [14]	China	Retrospective analysis	Non-severe	45	56 (39-67)	19 (42.2%)	4 (8.9%)
			Severe	67	68 (57-77)	38 (56.7%)	11 (16.4%)
Deng 2020 (B) [15]	China	Retrospective analysis	Survivor	212	62.25 (52-70)	97 (45.8%)	14 (6.6%)
			Non-survivor	52	74.5 (65.3-81.8)	33 (63.5%)	18 (34.6%)
Fan 2020 [16]	China	Retrospective analysis	Survivor	26	46.23 (12.01)	17 (65.4%)	0 (0%)
			Non-survivor	47	65.46 (9.74)	32 (68.1%)	7 (14.89%)
Forsblom 2021 [17]	Finland	Retrospective analysis	Survivor	517	56 (45-66)	269 (52.0%)	24 (4.6%)
			Non-survivor	68	79 (69-84)	47 (69.1%)	13 (19.1%)
Giannini 2021 [18]	Italy	Retrospective analysis	Survivor	882	64 (57-74)	584 (66.7%)	28 (4.9%)
			Non-survivor	211	77 (70-82)	158 (74.9%)	15 (16.9%)
Gu 2020 [19]	China	Retrospective analysis	Survivor	181	62.4 (14.7)	117 (64.6%)	15 (8.3%)
			Non-survivor	94	70.7 (13.3)	56 (59.6%)	25 (26.6%)
Guan 2020 [20]	China	Retrospective analysis	Non-severe	926	45.0 (34.0–57.0)	537 (58.0%)	17 (1.8%)
			Severe	173	52.0 (40.0–65.0)	100 (57.8%)	10 (5.8%)
	India		Not-ICU	168	37.93±16.44	96 (57.1%)	6(3.57%)

Gupta 2020 [21]		prospective observational analysis	ICU	32	51.06±16.03	20 (62.4%)	3(9.38%)
Gupta 2021 [22]	USA	Retrospective analysis	Survivor	121	68 [58, 80]	66 (54.5%)	14 (11.6%)
			Non-survivor	59	71 [61, 83]	32 (54.2%)	11 (18.6%)
Hewitt 2020 [23]	UK	observational cohort study	Survivor	1,139	NS	648 (71·8%)	213 (61·7%)
			Non-survivor	425	NS	255 (28·2%)	132 (38·3%)
Huang 2020 [24]	China	Retrospective analysis	Survivor	283	52.5 ± 16.6	149 (52.7%)	11 (3.9%)
			Non-survivor	16	69.2 ± 9.7	11 (68.8%)	2 (12.5%)
Iaccarino 2020 [25]	Italy	Observational Study	Survivor	1,403	64.7±0.4	892 (63.6%)	160 (11.4%)
			Non-survivor	188	79.6±0.8	125 (66.5%)	56 (29.8%)
Islam 2020 [26]	Bangladesh	Retrospective analysis	Survivor	991	NS	632 (63.8%)	4 (16.0%)
			Non-survivor	25	NS	19 (76.0%)	36 (3.6%)
Kuno 2020 [27]	USA	Retrospective analysis	Survivor	7,185	NS	NS	505 (7.0%)
			Non-survivor	723	NS	NS	218 (30.2%)
Lagi 2020 [28]	Italy	Retrospective analysis	Not-ICU	68	62 (50-72)	41 (60.3%)	7 (10.3%)
			ICU	16	67 (58-71)	14 (87.5%)	5 (31.3%)
Lee 2020 [29]	Korea	Retrospective analysis	Non-severe	557	52.10 (18.29)	155 (27.8%)	11 (2.9%)
			Severe	137	71.41 (13.17)	57 (41.6%)	6 (5.9%)
Lendorf 2020 [30]	Denamrk	Retrospective analysis	Not-ICU	91	69 (57-79)	50 (54.9%)	16 (17.6%)
			ICU	20	64 (53.72)	17 (85.0%)	3 (15.0%)
Levy 2020 [31]	USA	Retrospective analysis	Survivor	4,048	NS	2321 (57.3%)	270 (7.1%)
			Non-survivor	1,185	NS	736 (62.1%)	184 (16.5%)
Li 2020 [32]	China	Retrospective analysis	Non-severe	16	NS	6 (37.5%)	3 (18.8%)
			Severe	9	NS	6 (66.7%)	1 (11.1%)
			Survivor	20	NS	8 (40.0%)	3 (15.0%)
			Non-survivor	5	NS	4 (80.0%)	1 (20.0%)
Li 2020 (B) [33]	China	Retrospective analysis	Survivor	60	62 (53-70)	33 (55.0%)	2 (3.3%)
			Non-survivor	14	71 (69-77)	11 (78.6%)	4 (28.6%)
Liu 2020 [34]	China	Retrospective analysis	Non-severe	1,087	57.75 (3.5)	475 (43.7%)	79 (7.3%)
			Severe	957	64.9 (3.8)	525 (54.9%)	120 (12.5%)
Liu 2020 (B) [35]	China	Retrospective analysis	Non-severe	61	51(20-83)	31 (50.8)	2 (3.3%)
			Severe	23	67(31-91)	16 (69.6%)	6 (26.1%)
Loffi 2020 [36]	Italy	Retrospective analysis	Survivor	1,000	NS	NS	69 (6.0%)
			Non-survivor	252	NS	NS	55 (21.9%)
Maeda 2020 [37]	USA	Retrospective analysis	Not-ICU	167	65 [51, 77]	96 (57.5%)	27 (16.2%)
			ICU	57	67 [58, 76]	31 (54.4%)	18 (31.6%)
Mousseaux 2021 [38]	France	prospective observational cohort	Non-severe	42	69.3 (12.5)	24 (57.1%)	0 (0.0%)
			Severe	63	76.1 (11.3)	50 (79.4%)	1 (2.4%)
Patel 2021 [39]	USA	Retrospective analysis	Survivor	696	61 (47–73)	384 (55.2%)	131 (18.8%)
			Non-survivor	106	73 (61–84)	67 (63.2%)	31 (29.2%)
Peterson 2021 [40]	USA	Retrospective analysis	Survivor	275	NS	NS	53 (19.3%)
			Non-survivor	80	NS	NS	24 (30.0%)
Qin 2021 [41]	China	Retrospective analysis	Survivor	239	63 (52–69)	113 (47.3%)	22 (9.2%)
			Non-survivor	23	69 (65–74)	10 (43.5%)	4 (17.4%)
	Italy		Survivor	157	65.6 (15.6)	95 (60.5%)	16 (10.2%)

Russo 2020 [42]		Retrospective analysis	Non-survivor	35	77.0 (8.3)	20 (57.1%)	10 (28.6%)
Scoccia 2021 [43]	Italy	Retrospective analysis	Survivor	1,239	NS	NS	792 (58.8%)
			Non-survivor	386	NS	NS	329 (85.2%)
Serin 2020 [44]	Turkey	Retrospective analysis	Survivor	2,149	NS	NS	147 (6.8%)
			Non-survivor	68	NS	NS	18 (26.5%)
Shang 2020 [45]	China	Retrospective analysis	Survivor	64	62 (53-71)	37 (57.8%)	8 (12.5%)
			Non-survivor	49	73 (64.5-79)	36 (73.5%)	20 (40.8%)
Shi 2020 [46]	China	Retrospective analysis	Survivor	609	61 (49–70	287 (47.1%)	39 (6.4%)
			Non-survivor	62	74 (66–81)	35 (56.5%)	21 (33.9%)
Singh 2021 [47]	Netherlands	Retrospective analysis	Non-severe	418	67.19±15.34	214 (51.19%)	49 (19.76%)
			Severe	1,166	69.3 (12.8)	723 (62.0%)	112 (9.6%)
			Survivor	1,209	66.2 (13.7)	686 (56.7%)	105 (8.7%)
			Non-survivor	375	77.10±8.61	251 (67.43)	56 (21.70%)
Slipczuk 2021 [48]	USA	Retrospective analysis	Survivor	296	72 (64–80)	96 (48.7%)	131 (44.3%)
			Non-survivor	197	68 (57–76)	148 (50.0%)	108 (54.8%)
Sun 2020 [49]	China	Retrospective analysis	Survivor	123	67 (64-72)	51 (41.5%)	15 (12.2%)
			Non-survivor	121	72 (66-78)	82 (67.8%)	20 (16.7%)
Sun 2020 (B) [50]	China	Retrospective analysis	Non-severe	310	48 (35-62)	157 (50.6%)	12 (3.9%)
			Severe	26	65 (63-75)	20 (76.9%)	5 (19.2%)
Terlecki 2021 [51]	Poland	Retrospective analysis	Survivor	1,506	61.0 (49–72)	757 (50.3%)	223 (14.8%)
			Non-survivor	223	78 (69–84)	129 (57.8%)	67 (30.0%)
Turagam 2020 [52]	USA	Retrospective analysis	Survivor	88	58 (45–71)	64 (73%)	22 (25%)
			Non-survivor	52	71 (58–78)	38 (73%)	13 (25%)
Wang 2020 [53]	China	Retrospective analysis	Survivor	277	46.0 (14.4)	129 (46.6%)	5 (1.8%)
			Non-survivor	19	65.6 (12.6)	11 (57.9%)	5 (26.3%)
Wei 2020 [54]	China	Retrospective analysis	Non-severe	262	50.0 (39.0–57.0)	NS	8 (5.2%)
			Severe	14	65.0 (60.0–72.8)	NS	4 (28.6%)
Xie 2020 [55]	China	Retrospective analysis	Non-severe	38	60.1 (7.5)	14 (36.8%)	1 (2.6%)
			Severe	24	71.2 (6.6)	13 (54.2%)	6 (25.0%)
Xiong 2020 [56]	China	Retrospective analysis	Non-severe	61	56.0 (37.0–64.0)	42 (68.9%)	4 (6.6%)
			Severe	55	64.0 (53.0–76.0)	38 (69.1%)	13 (23.6%)
Xu 2020 [57]	China	Retrospective analysis	Non-severe	47	52.49 (14.62)	21 (44.68%)	3 (17.65%)
			Severe	41	63.2 (14.5)	20 (48.8%)	4 (9.8%)
Yuan 2020 [58]	China	Retrospective analysis	Non-severe	61	66 (52, 69)	30 (49.2%)	4 (6.6%)
			Severe	46	68 (61, 76)	21 (45.7%)	5 (10.9%)
Zhang 2020 [59]	China	Retrospective analysis	Non-severe	182	49.26 (16.13)	75 (41.2%)	5 (2.7%)
			Severe	359	61.9 (15.4)	180 (50.1%)	36 (10.0%)
Zhao 2020 [60]	China	Retrospective analysis	Non-severe	414	54 (37–65)	71 (56.8%)	17 (4.1%)
			Severe	125	70 (61.5–80)	184 (44.4%)	20 (16.0%)
Zhou 2020 [61]	China	Retrospective analysis	Survivor	137	52 (45-58)	81 (59.1%)	2 (1.5%)
			Non-survivor	54	69 (63-76)	38 (70.4%)	13 (24.1%)
Zou 2020 [62]	China	Retrospective analysis	Survivor	102	56.45 ± 12.64	47 (46.08%)	5 (4.90%)
			Non-survivor	52	68.98 ± 9.22	20 (38.46%)	18 (34.62%)

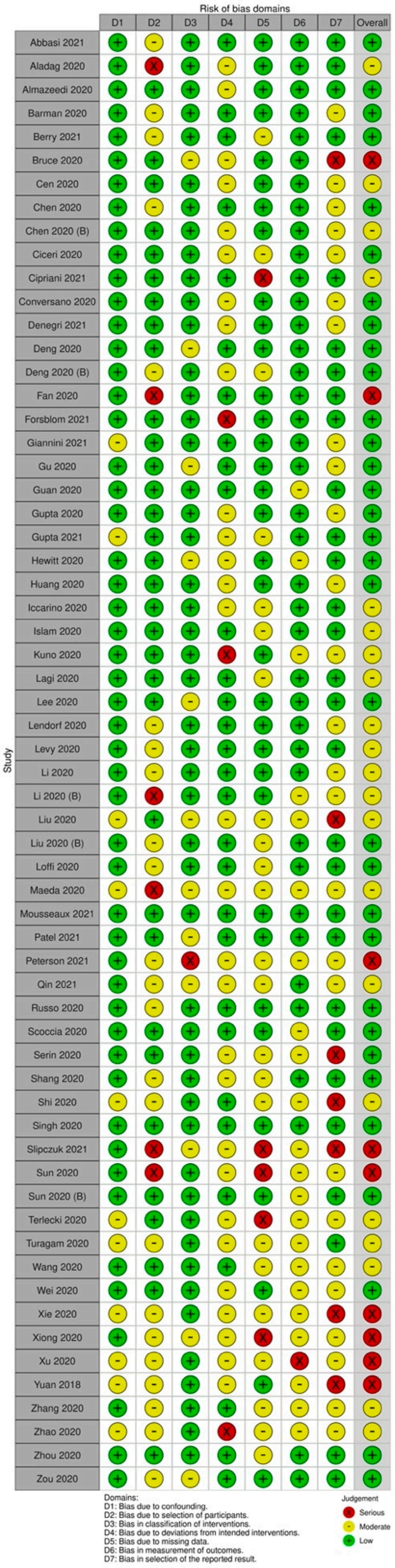

Figure S1. A summary table of review authors' judgements for each risk of bias item for each study.

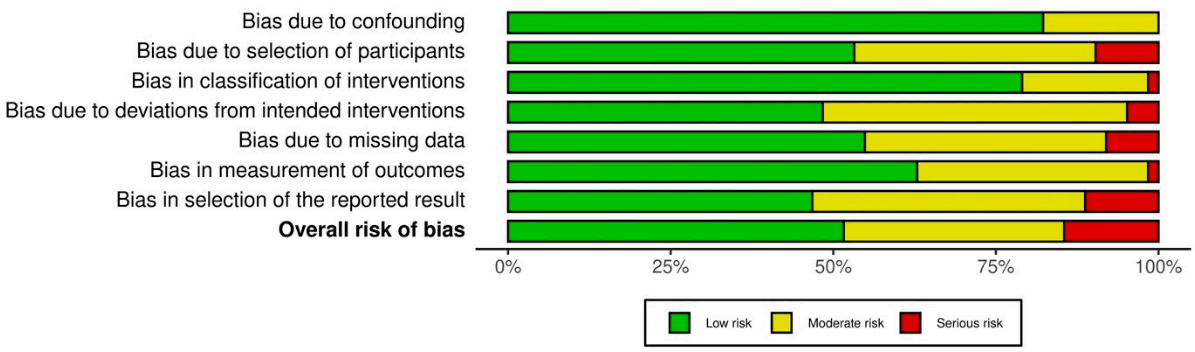

Figure S2. A plot of the distribution of review authors' judgements across studies for each risk of bias item.
